# Carbon Monoxide Induces Heme Oxygenase-1 to Modulate STAT3 Activation in Endothelial Cells via S-Glutathionylation

**DOI:** 10.1371/journal.pone.0100677

**Published:** 2014-07-29

**Authors:** Yan-Chang Yang, Yu-Ting Huang, Chia-Wen Hsieh, Po-Min Yang, Being-Sun Wung

**Affiliations:** 1 Department of Microbiology, Immunology and Biopharmaceuticals, National Chiayi University, Chiayi, Taiwan, ROC; 2 Department of Ophthalmology, Chiayi Christian Hospital, Chiayi, Taiwan, ROC; University of Illinois at Chicago, United States of America

## Abstract

IL-6/STAT3 pathway is involved in a variety of biological responses, including cell proliferation, differentiation, apoptosis, and inflammation. In our present study, we found that CO releasing molecules (CORMs) suppress IL-6-induced STAT3 phosphorylation, nuclear translocation and transactivity in endothelial cells (ECs). CO is a byproduct of heme degradation mediated by heme oxygenase (HO-1). However, CORMs can induce HO-1 expression and then inhibit STAT3 phosphorylation. CO has been found to increase a low level ROS and which may induce protein glutathionylation. We hypothesized that CORMs increases protein glutathionylation and inhibits STAT3 activation. We found that CORMs increase the intracellular GSSG level and induce the glutathionylation of multiple proteins including STAT3. GSSG can inhibit STAT3 phosphorylation and increase STAT3 glutathionylation whereas the antioxidant enzyme catalase can suppress the glutathionylation. Furthermore, catalase blocks the inhibition of STAT3 phosphorylation by CORMs treatment. The inhibition of glutathione synthesis by BSO was also found to attenuate STAT3 glutathionylation and its inhibition of STAT3 phosphorylation. We further found that HO-1 increases STAT3 glutathionylation and that HO-1 siRNA attenuates CORM-induced STAT3 glutathionylation. Hence, the inhibition of STAT3 activation is likely to occur via a CO-mediated increase in the GSSG level, which augments protein glutathionylation, and CO-induced HO-1 expression, which may enhance and maintain its effects in IL-6-treated ECs.

## Introduction

IL-6 is a key proinflammatory cytokine involved in a wide spectrum of diseases, including atherosclerosis, osteoporosis, arthritis, diabetes and certain cancers [1]. IL-6 signaling has now been extensively studied and is known to be mediated through a tyrosine kinase mechanism involving the JAK/STAT pathway [1]. STAT3 is a member of a family of functionally related STAT proteins (signal transducers and activators of transcription) and plays a key role in a variety of biological activities, including cell growth and differentiation, inflammation and immune responses [2]. Tyrosine phosphorylation of STAT3 results in the homo- or heterodimerization of this protein and its subsequent translocation into the nucleus where these dimeric complexes bind to target response elements, including interferon response elements (IREs) and serum-inducible elements (SIEs), and activate gene expression [3]. Indeed, we and others have demonstrated that STAT3 is a redox sensitive transcriptional factor that is functionally modulated by the intracellular redox state [4]–[6].

Heme-oxygenase-1 (HO-1) is a critical protein in the response to oxidative stress and its main function is associated with the degradation of heme to biliverdin, iron, and carbon monoxide (CO) [7]. Recent studies have demonstrated that HO-1 functions as part of the cytoprotective mechanisms that underlie antioxidant activities [8] and shows potential as a novel therapeutic target for cardiovascular diseases [9]. CO has been found to mediate many biological functions, such as anti-inflammation, proliferation arrest, and vasodilatation [10], and has shown considerable potential in therapeutic applications [11]. However, the detailed mechanisms underlying CO induced cytoprotective effects remain unclear.

Cytochrome c oxidase acts as the mitochondrial enzyme responsible for the reduction of oxygen into water as the final step of the electron transport chain. NO has long been known to increase ROS via the inhibition of cytochrome *c* oxidase function by competing with oxygen binding and thereby alter the cellular redox state [12]. Previous studies further demonstrated that through CO binding cytochrome c oxidases decrease oxygen levels and increases low levels of ROS levels [13], [14]. We reported previously that low levels of ROS may enhance protein glutathionylation by increasing the intracellular GSSG level [15]. Protein glutathionylation plays a critical role in the regulation of thiol/disulfide homeostasis within the cell and functions in protecting cysteine residues from irreversible oxidative damage [16]. A considerable body of evidence has now demonstrated that the glutathionylation of protein cysteine thiols regulates protein function [17], suggesting that glutathionylation is a physiologically important mechanism for controlling the activation of key signaling pathways. Recent studies have also found that STAT3 can be glutathionylated under treatment with GSSG or phytochemicals, which reduces the GSH/GSSG ratio, induces the post-translational modification of cysteine residues on the STAT3 protein and inhibits phosphorylation [18]; [19]. Thus, we speculated that glutathionylation plays a role in CO-modulated STAT3 activation.

Recently, metal carbonyls have been found to have the ability to release CO and provoke the same physiological reactions as CO, leading to their designation as “carbon monoxide-releasing molecules” (CO-RMs) [20]. CORMs have been shown to act pharmacologically by mimicking the bioactive effects attributed to HO-1 and CO [21], [22]. Previous studies have identified that CORM-released CO attenuates STAT3 activation and therefore suppresses inflammation in macrophages [23]. Our recent finding demonstrated CORMs induce protein S-glutathionylation to modulate NF-κB activity in ECs [24]. However, little detail is yet known regarding the mechanism of CORM-modulated STAT3 pathway in endothelial cells (ECs). In our present study, we investigated the effects and the mechanisms underlying the activities of the CORMs tricarbonyl dichlororuthenium (II) dimer (TCDC) and CO-releasing agent methylene chloride (MC) in ECs. We have elucidated that CO-induced STAT3 glutathionylation is a mechanism that enhances the suppression of STAT3 activation.

## Materials and Methods

### Materials

Bacterially derived IL-6 was purchased from Calbiochem (San Diego, CA). Homo sapiens heme oxygenase-1 expression vector (HMOX1) was obtained from OriGene (Rockville, MD). Antibodies against both native STAT3 and STAT3 phosphorylated on tyrosine 705 or serine 725 were obtained from Santa Cruz Biotechnology (Santa Cruz, CA). ECL reagents were purchased from Pierce (Rockford, IL). Luciferase assay kits were purchased from Promega (Madison, WI). Peroxidase-conjugated anti-rabbit and anti-mouse antibodies were obtained from Amersham (Arlington Heights, IL) and nitrocellulose was obtained from Schleicher & Schuell (Dassel, Germany). Methylene chloride (MC) was purchased from J.T. Baker (Phillipsburg, NJ). Tricarbonyl dichlororuthenium (II) dimer (TCDC) was purchased from Sigma (St. Louis, MO). All other reagents were purchased from Sigma (St. Louis, MO) and Merck (Darmstadt, Germany).

### Endothelial cell cultures

Bovine aortic endothelial cells (BAECs) were isolated from whole bovine aortas as previously described by Gospodarowicz [25] with modifications. Briefly, aortas from a slaughterhouse of Chyayi city meat market (Chyayi, Taiwan) were harvested and washed in PBS, and their lumenal surfaces were exposed by incubation with 0.1% collagenase for 10 min. Endothelial cells were then isolated and cultured in Dulbecco's modified Eagle's medium (DMEM; Invitrogen, Carlsbad, CA) supplemented with 10% fetal bovine serum (FBS; Invitrogen), 100 U/mL penicillin and 100 µg/mL streptomycin. Cells were maintained at 37°C in a humidified atmosphere of air and 5% CO_2_ and grown in Petri dishes for three days to reach confluence [26]. The culture medium was then replaced with serum free DMEM and the cells were incubated for 12 h prior to experimental treatments.

### Cell viability assay

Cell viability was measured using an Alamar blue assay (Serotec, Oxford, UK) in accordance with the manufacturer's instructions. The assay is based on the detection of metabolic activity in living cells using a redox indicator that changes from an oxidized (blue) to a reduced (red) form. The intensity of the red color is proportional to the viability of the cells, and is calculated as the difference in the absorbance values at 570 and at 600 nm and expressed as a percentage of the control.

### Measurement of intracellular ROS

Cells were cultured at 37°C in the presence or absence of the reagents indicated in the figures, washed with PBS and then incubated with 20 µM of the peroxide sensitive fluorescent probe 5-(and-6)-carboxy-2,7,dichlorodihydro fluorescein diacetate (carboxy-H_2_DCFDA; Molecular Probes, Eugene, OR) for an additional 30 min at 37°C. After two further washes with PBS, the cells were solubilized with 1% SDS and 5 mM Tris HCl (pH 7.4). Fluorescence was measured by spectrofluorophotometry (Shimadzu, Rf-5301PC) with excitation and emission wavelengths of 450 nm and 520 nm, respectively. Samples were assayed in triplicate.

### Chemiluminescence assay

Superoxide levels were measured by lucigenin-amplified chemiluminescence. ECs were lysed with a buffer containing lucigenin (200 µM) as previously described [27]. Measurements were initiated upon the addition of lysis buffer and recorded using a luminometer (Berthord, Pforzheim, Germany).

### GSH Assay

GSH levels were determined using the method originally described by Kamencic [28]. Briefly, cells were cultured at 37°C in the presence or absence of specific treatment reagents, as indicated in the figures, washed twice with PBS and then incubated with monochlorobimane (MCB, 2 mM) in the dark for 20 min at 37°C. After two further washes with PBS, the cells were solubilized with 1% SDS and 5 mM Tris HCl (pH 7.4]. Fluorescence was measured by spectrofluorophotometry (Shimadzu, Rf-5301PC) with excitation and emission wavelengths of 380 and 470 nm, respectively. The assay for detecting GSH levels in vitro was performed identically but without cell lysates. The levels of intracellular GSH were quantified using a GSH solution as a standard.

### Determination of GSSG level

Level of GSSG was determined using the method by reduce the GSSG to GSH. Briefly, ECs were harvested after the respective treatment, washed twice and suspended in cold PBS. The lysate was treated with 4-vinylpyridine to deplete GSH. 4-vinylpyridine was added to a final concentration of 0.1% (v/v) and then incubated for 1 h at room temperature. At this concentration, 4-vinylpyridine is able to react with all GSH without interfering with GSSG determination. After centrifugation supernatant incubates with 0.1M sodium phosphate buffer containing 25U/ml glutathione reductase. GSSG determination follows the GSH assay using fluorescent probe MCB.

### Detection of protein glutathionylation using BioGEE

Biotin-labeled glutathione ester (BioGEE, G36000, Invitrogen) is a cell permeable biotinylated GSH that can detect proteins that form adducts to reactive thiols using an avidin-agarose pull down experiment. These biotinylated proteins can then be observed by SDS-PAGE [29]. The BioGEE mixture is added to cell culture medium at a final concentration of 100 µM. At designated time points, cell lysates are prepared, pre-cleared with agarose beads and then incubated with streptavidin-conjugated agarose beads (100 µl/mg of protein) for 30 min at 4°C to specifically bind protein–BioGEE complexes. After centrifugation and washing, the beads are incubated for 30 min with 10 mM DTT in PBS/EDTA/SDS to elute proteins. Total glutathionylated proteins are then resolved by SDS-PAGE and detected by silver staining.

### Promoter constructs and luciferase assays

An 3xSTAT3/Luc fragment containing tandem repeats of double-stranded oligonucleotides spanning the STAT3 binding site (5′-CATTTCCCGTAAATC-3′) [30] was amplified with the primers sense: 5′-CATT TCCCGTAAAT CCATTTCCCGTAAATCCATTTCCCGTAAATC-3′ and then introduced into the pGL3 promoter plasmid (Promega, Madison, WI). All transfection experiments were performed using Lipofectamine 2000 reagent (Invitrogen) in accordance with the manufacturer's instructions. For luciferase assays, the cell lysate was first mixed with luciferase substrate solution (Promega), and the resulting activity was measured using a luminometer. For each experiment, luciferase activity was determined in triplicate and normalized using β-galactosidase activity.

### siRNA targeting of HO-1

A human HO-1 siRNA 5'-CUGUGUCCCUCUCUCUGGA-3' (Sigma, St. Louis, MO); and a control siRNA, 5-GCAAGCUGACCCUGAAGUUCAU-3 (Ambion, Austin, TX) were used to target the HO-1 gene. The effects of these siRNA molecules have been described in our previous study [31].

### Preparation of cytosolic and nuclear lysates

To separate cytosol from nuclear proteins, ECs were collected by scraping in cold PBS. The cell pellet was then lysed in 10 mM HEPES, 1.5 mM MgCl_2_, 10 mM KCl, 0.5 mM DTT, 0.5 mM PMSF and 0.3% nonidet P-40. After 5 min of centrifugation at 1500 *g* at 4°C, the supernatant was collected and designated as the cytosolic fraction. Nuclear proteins were then extracted using a buffer containing 25% glycerol, 20 mM HEPES, 0.6 M KCl, 1.5 mM MgCl_2_ and 0.2 mM EDTA. Protein concentrations were determined using a protein assay DC system (Bio-Rad, Richmond, CA).

### Western blotting

Whole lysates of ECs were prepared as previously described [32]. A total of 1×10^6^ cells were lysed on ice in lysis buffer (1% NP-40, 0.5% sodium deoxycholate, 0.1% SDS and a protease inhibitor mixture) and whole-cell extracts were boiled for 5 min prior to separation on 10% SDS-PAGE, in which the protein samples were evenly loaded. The proteins were then transferred to a nitrocellulose filter (Millipore, Bedford, MA) in Tris-glycine buffer at 10 V for 1.5 hours. The membranes were then blocked with PBS containing 5% nonfat milk and incubated with antibodies for two hours at 4°C, with gentle shaking. The results were visualized by chemiluminescence using ECL in accordance with the manufacturer's instructions. Relative protein levels were determined by scanning densitometry analysis using the Uni-photo band tool (EZ lab, Taiwan, ROC). All of the values subjected to statistical analysis were normalized to internal control values (tubulin or actin) and were determined from at least three independent experiments.

### Immunoprecipitation

BAECs were washed with PBS and lysed in a non-reducing lysis buffer (1% NP-40, 0.5% sodium deoxycholate, 0.1% SDS and protease inhibitors). Protein cell lysates (300 µg) were cleared of abundant proteins by pre-incubation for one hour with Protein G plus-agarose. The supernatant was then collected and incubated with anti-p65 monoclonal antibodies for one hour at 4°C. Protein G plus-agarose was then added overnight at 4°C, and the mixture was then washed three times with PBS. The immunoprecipitated proteins were eluted and western blotting in a non-reducing buffer for the detection of STAT3 or GSH was performed using the corresponding antibodies.

### Statistical analysis

Values are expressed as the means ± SEM of at least three experiments. Statistical analyses were performed using ANOVA with the Tukey's *post-hoc* test (SPSS 12.0 software package, Chicago, IL). A confidence limit of *P*<0.05 was considered to be significant.

## Results

### The inhibition of IL-6-induced STAT3 activation by CO

**Figure 1:**
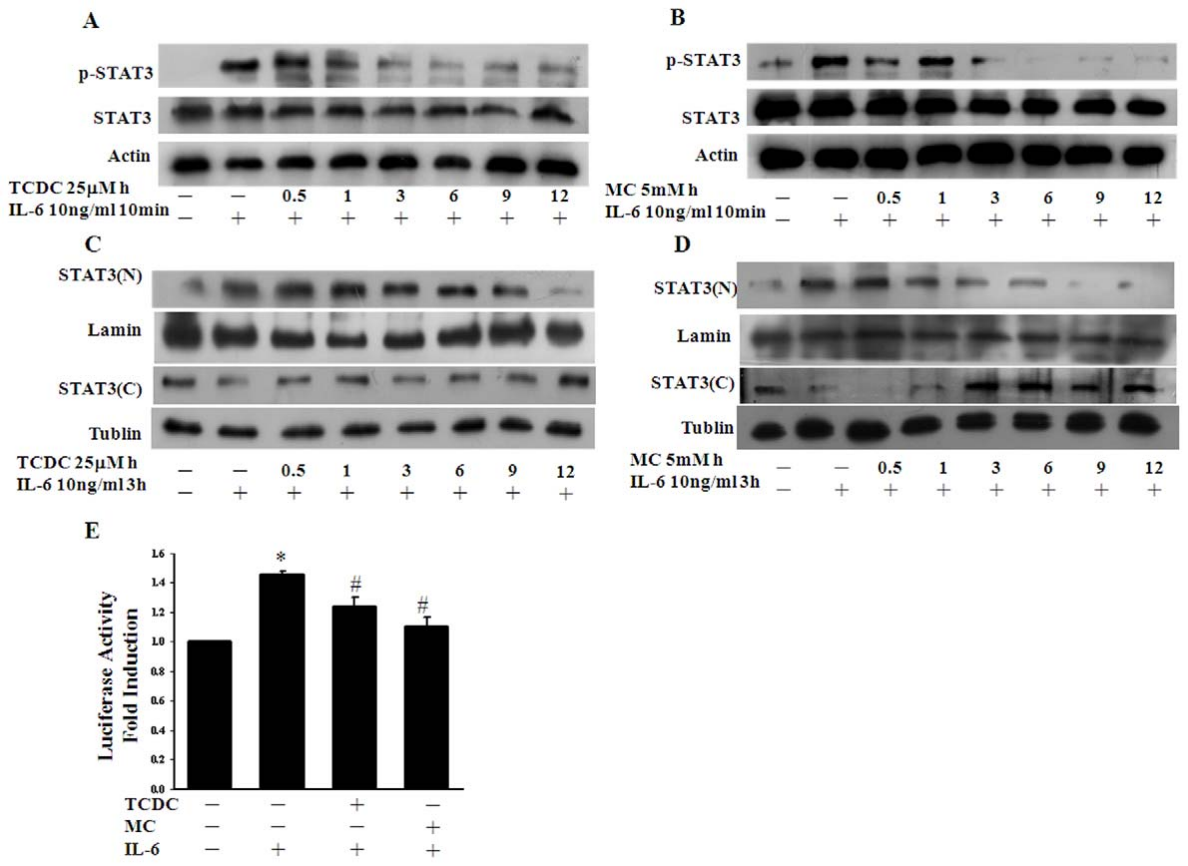
CO inhibition of IL-6-induced STAT3 activation. **A.** and **B.** EC cultures were induced by IL-6 treatment (10ng/ml) for 10 min and a portion of these cells were then pretreated with 25 µM TCDC or 5mM MC for the indicated times. Cell lysates were prepared and subjected to western blot analysis with antibodies against pTyr-STAT3 or STAT3 as indicated. The actin band intensities indicate equal loading of each well. **C.** and **D.** ECs were pretreated with TCDC or MC for the indicated times and then stimulated with IL-6. Nuclear (N) and cytosolic (C) extracts were then prepared and subjected to western blot analysis using STAT3 antibodies. The tubulin and lamin band intensities indicate equal loading, respectively. **E.** ECs were co-transfected with the STAT3 luciferase reporter construct and β-galactosidase for 16 hours. Cells were then exposed to TCDC or MC for 12 hours and to IL-6 for another 6 hours. Luciferase activity was normalized against β-galactosidase activity; the untreated value was taken as 1. *P<0.05 compared with untreated ECs, ^#^ *P*<0.05 compared with IL-6 alone (mean ± SEM).

Previous studies have demonstrated that HO-1 functions in an anti-inflammatory pathway [9]. The CORMs have been shown to function by reproducing the biological effects of CO which derive from HO activity [10]. We therefore tested whether the CO donors TCDC and MC suppress IL6-induced STAT3 activation. To examine whether CO regulates the phosphorylation of STAT3 in IL-6-treated ECs, we pretreated these cells with TCDC or MC and analyzed the IL-6-induced phosphorylation of tyr705. As shown in Figure 1A and 1B, the CORMs displayed a time-dependent inhibition of the tyrosine phosphorylation of STAT3 in IL-6-treated ECs. Nevertheless, the half-life of TCDC is short (approximately 20 min), but the inhibition of IL6-induced STAT3 phosphorylation by the CORMs was found to be more efficient during a longer pretreatment. We next assessed the cytotoxic effects of a 24 hour incubation of ECs with TCDC and MC (Figure S1 A and B). As shown in figure S1, neither TCDC nor MC showed toxic effects in these cells at this working dosage. We additionally tested the effects of 0.5 to 12 hour pretreatments with TCDC or MC on nuclear translocation and observed inhibitory effects on this process after a 3 hour pretreatment (Fig. 1 C and D). We further tested whether these two CORMs inhibit IL-6-induced STAT3 activation at the transcriptional level. Following a pretreatment for 12 hours (Fig. 1E), we found using a luciferase reporter assay that IL-6-induced STAT3 activation was indeed inhibited by TCDC and MC. Taken together, our data demonstrate that CO inhibits STAT3 activation and its subsequent functional activities in ECs.

### CO induces HO-1 expression upon STAT3 phosphorylation

**Figure 2:**
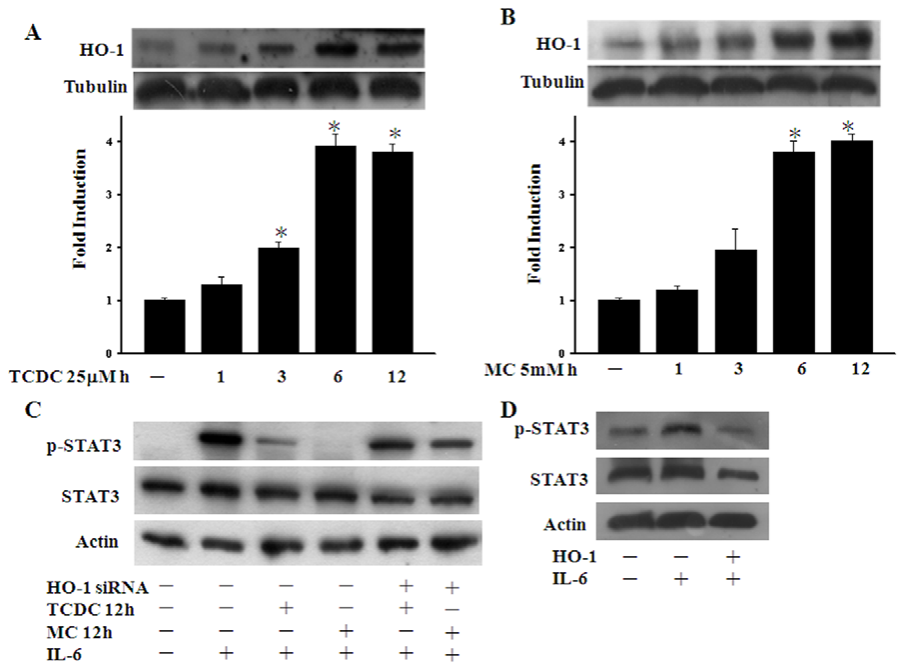
CO induces HO-1 expression upon STAT3 phosphorylation. **A.** and **B.** EC cultures were incubated with 25 µM TCDC or 5mM MC for the indicated periods. Western blotting analysis was then performed with antibodies against HO-1. **C.** ECs were transfected with control or HO-1 siRNA vectors for 36 hours and exposed to TCDC or MC for 12 hours. Cell lysates were subjected to western blot analysis with antibodies against pTyr-STAT3 or STAT3. **D.** ECs were transiently transfected with pcDNA vector or HO-1 plasmid. ECs transfected with pcDNA vector were exposed to IL-6 as a positive control. Cell lysates were subjected to western blot analysis with antibodies against pTyr-STAT3 or STAT3 as indicated. The quantification of the band intensities from three independent experiments was normalized to the control values (if the control was at near background, the background was set to 1). The resulting data are the mean ± SEM. *P<0.05 compared with untreated ECs.

Since the half-life of CORM-2 molecules is quite short, we speculated that the role of CORMs may be to trigger cytoprotective enzyme expression. Although CO is generated by HO-1, recent evidence also suggests that CO can induce HO-1 expression [21], [23]. We thud assayed the HO-1 levels in ECs during our CORM pretreatment time course and, as shown in Figure 2A and 2B, found that the HO-1 protein levels increased after 3 hours of CORM pretreatments and persisted for over 12 hours. Given the short half-life of the CORM-2 family members, we postulated that HO-1 may be involved in the lasting effects of these compounds. We therefore further investigated whether HO-1 could abolish the inhibition of STAT3 phosphorylation caused by TCDC and MC. ECs transfected with HO-1 siRNA were found to recover their CO-suppressed STAT3 phosphorylation levels after 12 hours of CORM treatment (Figure 2C). Furthermore, STAT3 phosphorylation was also found to be reduced by the overexpression of HO-1 in ECs (Figure 2D). As shown in figure S2, both HO-1 overexpression plasmid and hemin could induce HO-1 expression and HO-1 siRNA could abolish hemin-induced HO-1 expression. These results indicate that the HO-1 plays an important role in p65 glutathionylation following a 12 hours CORM treatment in ECs. Taken together, our findings indicate that CO-increased HO-1 mediates the long-term inhibitory effects of CORMs on STAT3 phosphorylation.

### CO increases oxidative stress in ECs

**Figure 3:**
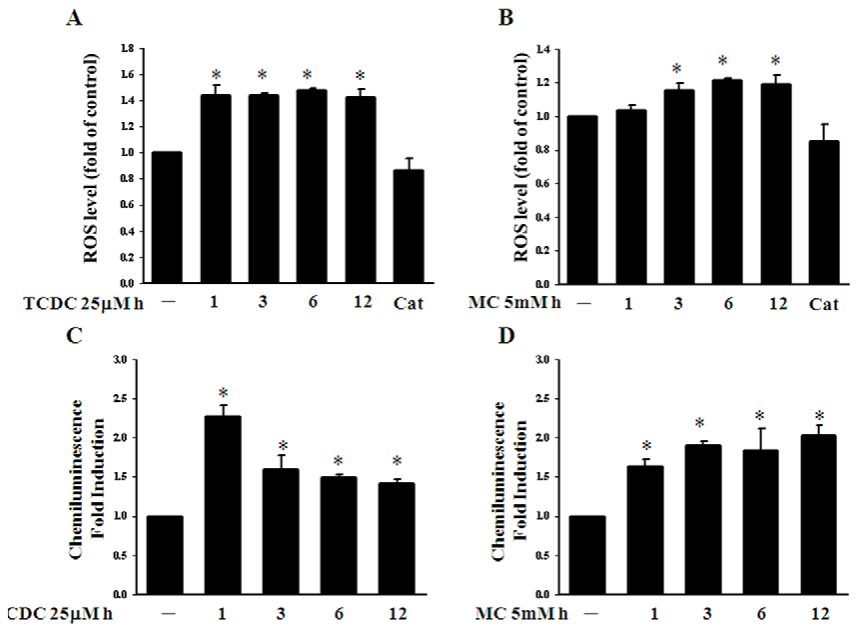
CO increases oxidative stress in ECs. **A.** and **B.** ECs were exposed to 25 µM TCDC or 5mM MC for 1, 3, 6 and 12 hours with or without pretreated 500U catalase for 30 min and the intracellular ROS levels were then measured. The results shown are the mean ± SEM. *P<0.05 compared with untreated ECs. **C. and D.** ECs were subjected to similar treatments prior to the measurement of intracellular ROS levels. The folds of lucigenin-amplified chemiluminescence are shown as the mean ± SEM. Results are presented as the mean ± SEM (n = 3). *P<0.05 compared with untreated ECs.

**Figure 4:**
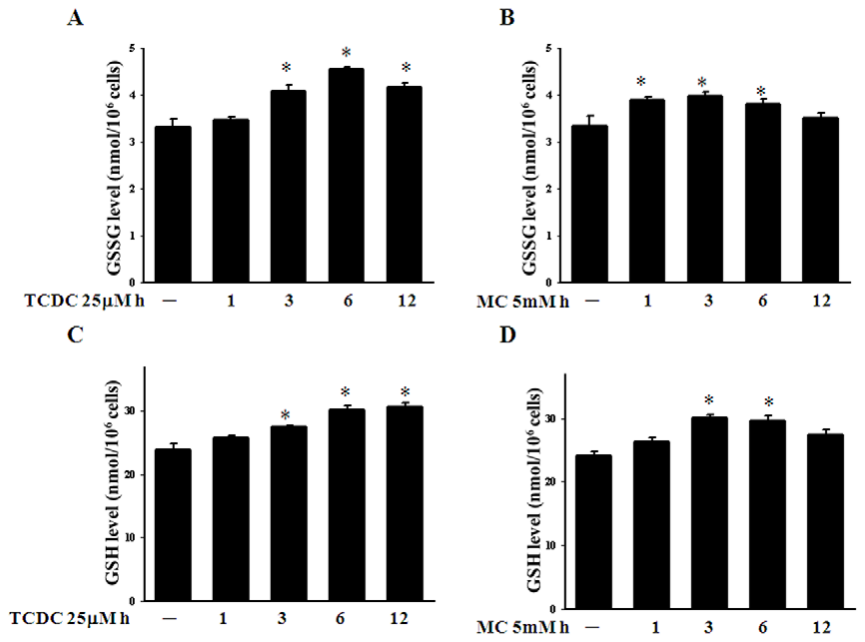
CO increases the GSSG level in ECs. **A.** and **B.** ECs were exposed to TCDC or MC for 1, 3, 6 and 12 hours and the intracellular SGGS levels were then measured. The results shown are the mean ± SEM. *P<0.05 compared with untreated ECs. **C. and D.** The intracellular GSH levels of ECs incubated with TCDC or MC for 1, 3, 6 and 12 hours. Results are presented as the mean ± SEM (n = 3). *P<0.05 compared with untreated ECs.

We have reported previously that HO-1 can be induced by oxidative stress [33]. Nevertheless, CO has long been known to increase low level ROS via the inhibition of cytochrome *c* oxidase by competing with oxygen binding [13], [14]. We thus examined intracellular ROS formation in CORM-treated ECs. As shown in Figure 3A and 3B, TCDC and MC can both increase the intracellular ROS level and superoxide levels (Figure 3C and D). CORM-treated ECs were pretreated with the anti-oxidant enzyme catalase and showed reduced ROS formation (Figure 3A and B). This may in turn enhance the GSSG level and induce protein glutathionylation, as demonstrated by our previous findings [15]. We therefore analyzed in our current study whether CO modulates the intracellular redox homeostasis in BAECs. As shown in Figure 4A and B, TCDC or MC increase the intracellular GSSG level after three hour of treatment. However, we also found that TCDC or MC increase the intracellular GSH level (Figure 4C and 4D). Although CORMs could induce a significant increase in GSSG, the GSH/GSSG ratio have different recovery time course after CORMs treatment. (Figure S3 A and B).

### The effects of CO on STAT3 glutathionylation

**Figure 5:**
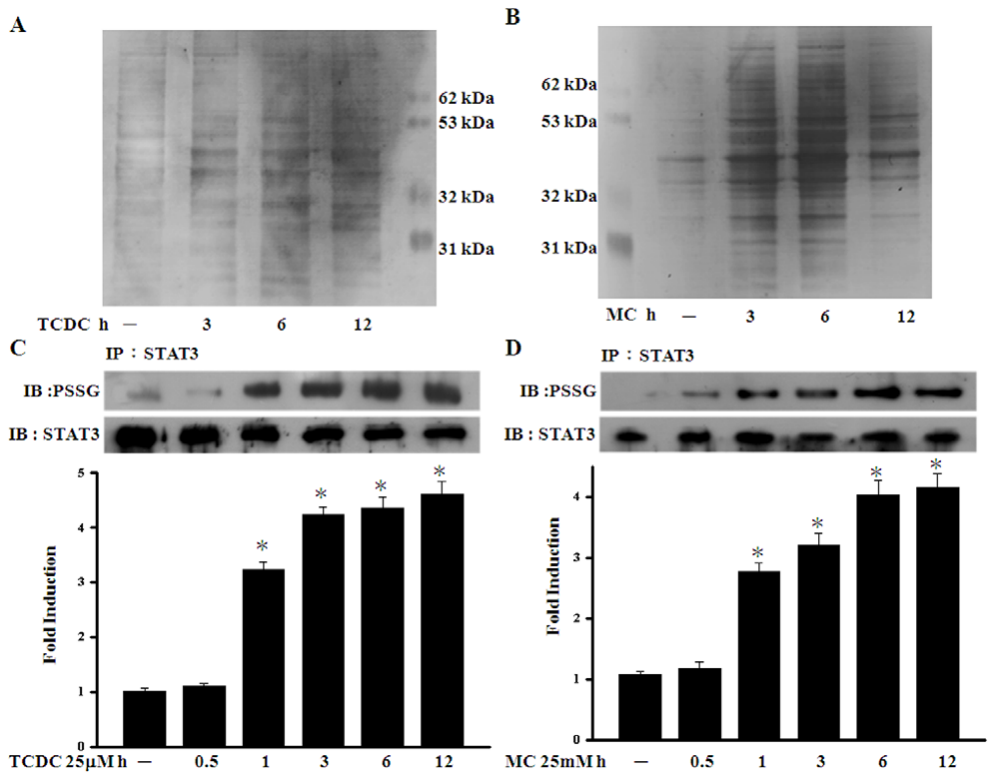
The effects of CO on STAT3 glutathionylation. **A.** and **B.** ECs were treated with 100 µM BioGEE for one hour and loaded with TCDC or MC for the indicated times to induce protein glutathionylation. **C.** and **D.** ECs were exposed to TCDC or MC for the indicated periods. S-glutathionylated STAT3 was immunoprecipitated and detected using an antibody against protein-SSG. The quantification of the band intensities was normalized to the control values. Results are presented as the mean ± SEM (n = 3). *P<0.05 compared with untreated ECs.

In our previous study, we found that low levels of ROS may enhance protein glutathionylation through an increased intracellular GSSG level [15]. Other previous studies have also demonstrated that glutathione disulfide (GSSG) induces the post-translational modification of cysteine residues in the STAT3 protein and inhibits STAT3 phosphorylation [18], [19]. To assess the effects of CO on protein glutathionylation, ECs were incubated with cell permeable biotinylated-glutathione-ethyl ester (Bio-GEE). Equal amounts of protein in each group were used to precipitate glutathionylated proteins using streptavidin agarose beads followed by resolution on SDS-PAGE. As shown in Figure 5A and 5B, silver stained SDS-PAGE gels indicated an increase in the total glutathionylated protein levels in CORM-treated samples as compared with untreated control cells. Using STAT3 antibody to detect the pull down proteins, we found TCDC and MC increase S-glutathionylation of STAT3 (Figure S4). We further found that the S-glutathionylation of STAT3 increased after one hour of TCDC and MC treatment in ECs and persisted for over 12 hours (Fig. 5C and 5D).

### STAT3 glutathionylation and phosphorylation are dependent on the ROS level in CO-treated ECs

**Figure 6:**
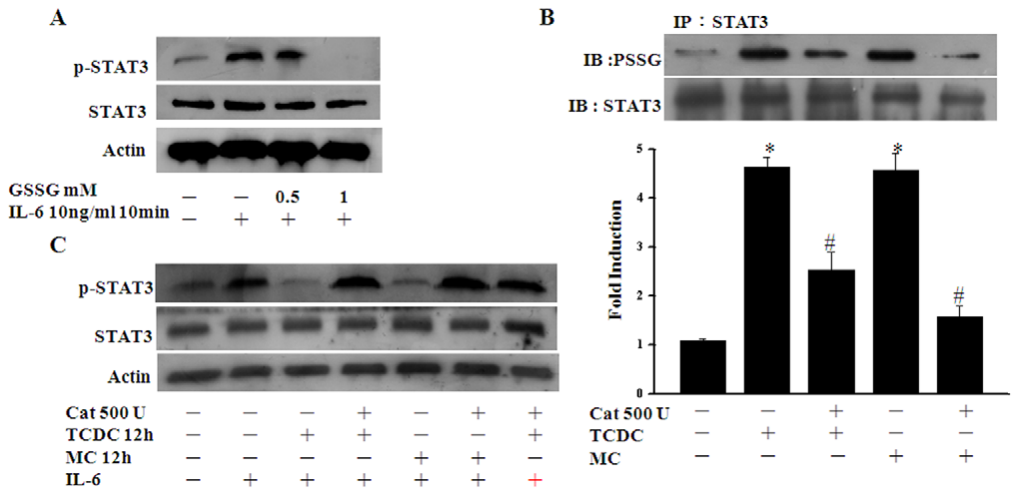
The STAT3 phosphorylation and glutathionylation levels are dependent on the ROS levels in CO-treated ECs. **A.** ECs subjected to IL-6 (10ng/ml) treatment for 10 min were pretreated with or without 0.5 or 1 mM GSSG for 30 minutes. Cell lysates were then subjected to western blotting with antibodies against pTyr-STAT3 or STAT3. **B.** ECs were pretreated with catalase at the indicated units for 30 min and then cultured without further treatment or exposed to TCDC or MC for 12 hours. S-glutathionylated STAT3 was then detected as above. The quantification of the band intensities was normalized to the control values. Results are presented as the mean ± SEM (n = 3). *P<0.05 compared with untreated ECs. ^#^ *P*<0.05 compared with TCDC or MC alone. **C.** ECs subjected to IL-6 treatment for 10 min were pretreated with 500 U catalase for 30 min and then cultured without further treatment or exposed to TCDC or MC for 12 hours. Cell lysates were then subjected to western blotting.

Given our finding that the GSSG levels increased over the same CORM pretreatment time, we further assessed the possible role of GSSG in STAT3 phosphorylation. As shown in Figure 6A, the levels of IL-6-induced STAT3 phosphorylation were suppressed after GSSG pretreatment. We therefore investigated whether CO-increased oxidative stress is required for CO-induced STAT3 phosphorylation. CORM-treated ECs were pretreated with the anti-oxidant enzyme catalase and showed reduced STAT3 glutathionylation (Fig. 6B). In a similar experiment, catalase was also found to suppress the inhibitory effects of CORMs upon STAT3 phosphorylation (Fig. 6C). These results indicate that both STAT3 glutathionylation and the inhibitory effects of CO require an increase in the intracellular ROS levels.

### The effects of CO are dependent on the intracellular GSH level

**Figure 7:**
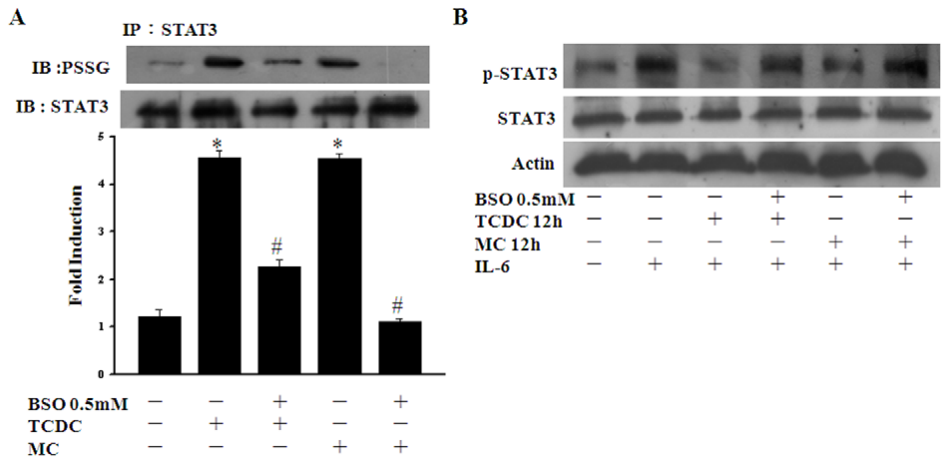
The effects of CO are dependent on the intracellular GSH level. **A.** ECs were pretreated with 0.5-glutathionylated STAT3 was then detected as described in Figure 5. *P<0.05 compared with untreated ECs. ^#^ *P*<0.05 compared with TCDC or MC alone. **B.** ECs subjected to IL-6 treatment for 10 min were pretreated with 0.5 mM BSO for 30 min and then cultured without further treatment or exposed to TCDC or MC for 12 hours. Cell lysates were then subjected to western blotting.

The total intracellular GSH level is particularly important for protein glutathionylation [34]. We therefore used 0.5 mM buthionine sulfoximine (BSO), a specific inhibitor of γ-glutamyl cysteine synthetase, to suppress GSH synthesis and test STAT3 glutathionylation and phosphorylation in CO-treated ECs. As shown in Figure 7A and 7B, ECs pretreated with BSO had a reduced STAT3 glutathionylation level and were refractory to the inhibitory effects of CORMs upon STAT3 phosphorylation.

### HO-1 modulates CO-induced STAT3 glutathionylation

**Figure 8:**
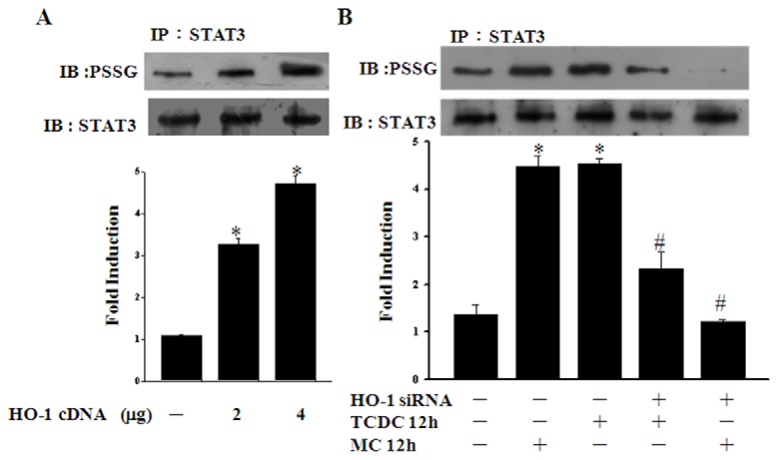
HO-1 modulates CO-induced STAT3 glutathionylation. **A.** ECs were transiently transfected with pcDNA vector or HO-1 plasmid. S-glutathionylated STAT3 was immunoprecipitated and detected using a protein-SSG antibody. *P<0.05 compared with untreated ECs. **B.** ECs were transfected with control or HO-1 siRNA vectors for 36 hours and then exposed to TCDC or MC for 12 hours. S-glutathionylated STAT3 was then detected as above. *P<0.05 compared with untreated ECs. ^#^ *P*<0.05 compared with TCDC or MC alone.

We further investigated whether HO-1 could increase the glutathionylation of STAT3. As shown in Figure 8A, STAT3 glutathionylation was increased by the overexpression of HO-1 in ECs. To then assess the mediating role of HO-1 in the effects of CO, we transfected ECs with HO-1 siRNA and found that this abolished STAT3 glutathionylation following a 12 hour CORM treatment (Fig. 8B). These results indicate that HO-1 plays an important role in STAT3 glutathionylation and suggest that CO-increased HO-1 mediates the long-term effects of CORMs.

## Discussion

STAT3 has been identified as a redox sensitive transcriptional factor and several lines of evidence now suggest that STAT3 activation is subject to redox regulation. Previous studies have reported that reactive oxygen species (ROS) trigger the tyrosine phosphorylation and nuclear translocation of STAT3 in human lymphocytes without cytokine stimulation [4]. In our previous studies, we pretreated ECs with chalcone or 15d-PGJ_2_, two electrophilic chemicals, and found that this attenuated the subsequent IL-6-induced phosphorylation of STAT3 [6], [35]. These inhibitory effects may come from electrophilic activity which may directly react with the thiol group in STAT3 or modulate the intracellular GSH level. Thus, we hypothesized that the oxidative modification of cysteine residues of STAT3 by S-glutathionylation may occur.

Another previous study has demonstrated S-glutathionylation markedly reduces STAT3 signaling in HepG2 cells [18], suggesting that the mechanism(s) underlying redox control may also regulate STAT3 signaling through the modification of cysteine thiol groups. In our current study, we report through the analysis of two CO donors, TCDC and MC, in ECs that there is a direct inhibition of STAT3 nuclear translocation and activation by protein S-glutathionylation. We find that IL-6-induced STAT3 signaling is attenuated by CO-increased low level oxidative stress, which could increase STAT3 protein S-glutathionylation in ECs. Furthermore, our analysis shows that CORMs induce HO-1 expression, which is required for the inhibition of STAT3 activation. Since the half-life of the CORMs is limited, we speculate that CORM-induced HO-1 may play a role in maintaining STAT3 glutathionylation at appreciable levels. It has been reported previously that CO donor attenuation or inhibition of IL-6-induced STAT3 activation is necessary for inducing the gene expression of pro-inflammatory molecules such as ICAM-1 [36]. This finding suggests that CORMs may have a positive regulatory effect on inflammatory states.

CO is generated endogenously during heme metabolism in a reaction catalyzed by HO enzymes. Recent studies have demonstrated that HO-1 functions as a novel therapeutic target for cardiovascular diseases [9]. The anti-inflammatory effects of HO-derived CO have been demonstrated to activate p38 MAPK and thereby induce IL-10 and the LPS-induced JNK pathway, and interfere with NF-κB signal transduction [37], [38]. In our present analyses, a significant increase in the HO-1 protein levels was found following CORM treatment of ECs for three hours (Fig. 2A and B). Indeed, upon transfection of ECs with HO-1 siRNA, CORMs failed to suppress either STAT3 nuclear translocation or STAT3 activation (Fig. 2D and E). The CO-induced HO-1 expression we observed in our experiments persisted for 12 hours and may provide an explanation for the long term effects of CORMs. These data also suggest that CO-induced HO-1 may augment and maintain the effects of CORMs in the inhibition of STAT3 activation. In addition, of the HO-1 products, CO and biliverdin, seem to be the major mediators of protective HO-1 effects. This protective property has been mainly attributed to bilirubin antioxidant activity [39]. However, in the present study we found that CORMs upregulate the ROS and GSGG level last to 12 hours (Fig. 3 and 4). Otherwise, in contrast to bilirubin, CO may play a major role regarding the protective mechanisms in BAECs. Our current data reveal that CO-induced STAT3 glutathionylation is such a mechanism, and this enhances our understanding of the protective effects of HO-1.

Previous studies have reported that CO stimulates the production of low levels of ROS via the inhibition of cytochrome c oxidase [13], [14]. The intracellular concentration of GSSG is crucial for redox homeostasis in ECs. In our present study, we found that CORMs upregulate the ROS and GSGG level (Fig. 3 and 4). In our previous studies, we have reported that an increased concentration of GSSG induces NF-κB p65 protein glutathionylation [15]. Hence, we demonstrate that CORM-induced STAT3 glutathionylation is dependent on an increased level of oxidative stress. In our current experiments, ECs treated with GSSG show increased STAT3 glutathionylation (Fig. 6A). However, pretreatment of these cells with the anti-oxidant enzyme catalase reduces STAT3 glutathionylation (Fig. 6B) and inhibits STAT3 activation (Fig. 6C). In addition, we used BSO (a selective inhibitor of γ-glutamylcysteine synthetase, the rate-limiting step in GSH synthesis) to reduce intracellular the glutathione level in ECs and found that this attenuated STAT3 glutathionylation (Fig 7A) and abrogated the inhibition of STAT3 phosphorylation by CORMs (Fig 7B). We contend from our findings that the anti-inflammatory properties of CO are mediated through GSSG and the intracellular GSH+GSSG level.

**Figure 9:**
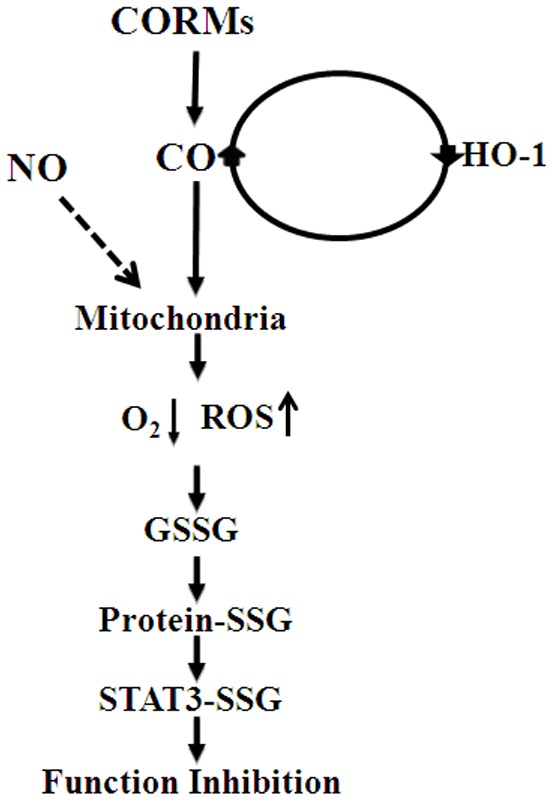
Proposed model of the inhibitory effects of CO upon STAT3 activation. NO and CO have long been known to increase ROS via the inhibition of cytochrome *c* oxidase function by competing with oxygen binding. The increased intracellular low levels oxidative stress induces protein S-glutathionylation to prevent STAT3 nuclear translocation under inflammatory cytokine treatment. See the “Discussion" section for details.

Atherogenesis is a chronic inflammatory response in which cytokines have been suggested to play a key role [40]. In our present study, we propose the model shown in Figure 9 to illustrate the inhibitory effects of CO upon IL-6-induced STAT3 activation. Our current finding that IL-6-induced STAT3 signaling is attenuated by CO and this inhibition is involved in HO-1 expression to modulate vascular disease conditions characterized in which pro-inflammatory signaling pathways. Furthermore, we demonstrate from our current findings that CO induces mildly oxidative physiological conditions, resulting in STAT3 glutathionylation and inhibition of its function. These cytoprotective effects of CO require the upregulation of HO-1 expression to enhance and maintain them over a long term.

## Supporting Information

Figure S1 **The cytotoxicity of CORMs in BAECs. A.** and **B.** ECs were incubated with TCDC or MC at the indicated concentrations for 24 hours and cell viability was measured spectrophotometrically using an Alamar blue assay. Data are the mean ± SEM of three independent experiments.
(TIF)

Figure S2 **HO-1 expression upon transfection of HO-1 siRNA and HO-1 cDNA.** BAECs were transfected with control, HO-1 siRNA or HO-1 plasmid for 36 hours and exposed to hemin for 12 hours. Western blotting analysis was then performed with antibodies against HO-1.
(TIF)

Figure S3 **The GSH/GSSG ratio under CORMs treatment.** **A.** and **B.** The GSH/GSSG ratio was determined at various time intervals. Results are presented as the mean from the data in Figure 4.
(TIF)

Figure S4 **The detect of STAT3 glutathionylation.** BAEC were loaded with biotin-labeled BioGEE (100 µmol/L, 1 h). Biotin-GSS tagged proteins were pulled-down with streptavidin-Sepharose beads and released with DTT (50 mmol/L), separated by SDS PAGE and immuno-blotted for STAT3.
(TIF)
