# Supplementary figures and images for: Carbon Monoxide Induces Heme Oxygenase-1 to Modulate STAT3 Activation in Endothelial Cells via S-Glutathionylation
Source: PLoS One. 2014 Jul 29;9(7):e100677. doi: 10.1371/journal.pone.0100677 (PMC4114553; doi:10.1371/journal.pone.0100677)

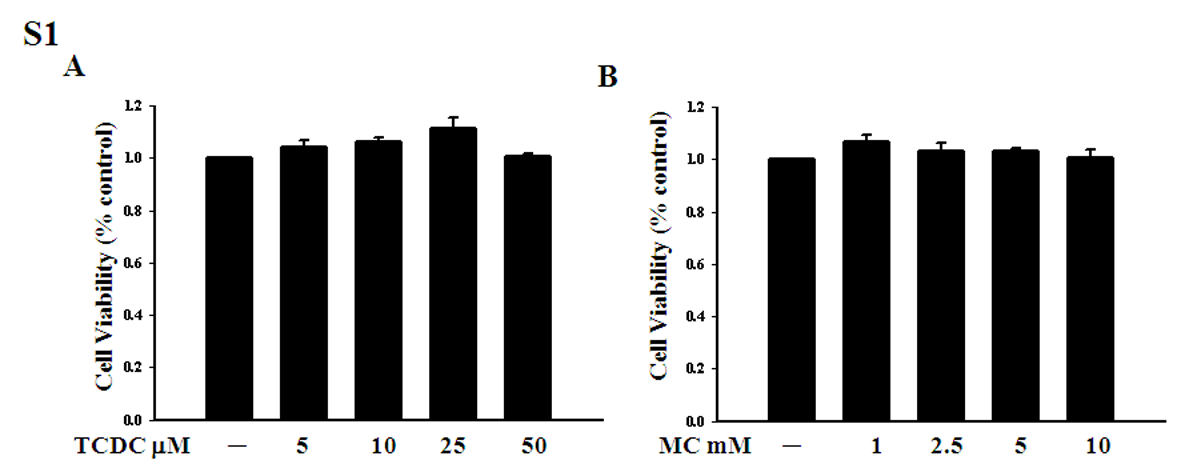

Supplement: Figure S1 — The cytotoxicity of CORMs in BAECs. A. and B. ECs were incubated with TCDC or MC at the indicated concentrations for 24 hours and cell viability was measured spectrophotometrically using an Alamar blue assay. Data are the mean ± SEM of three independent experiments. (TIF) [file pone.0100677.s001.tif]

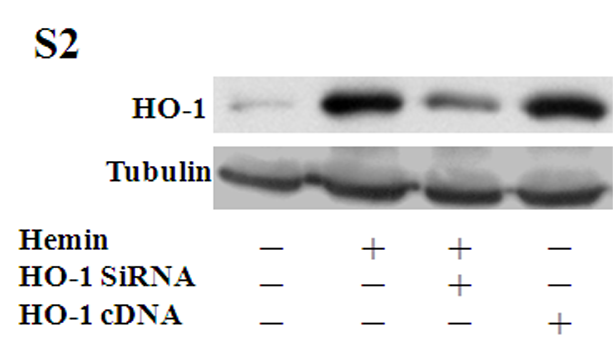

Supplement: Figure S2 — HO-1 expression upon transfection of HO-1 siRNA and HO-1 cDNA. BAECs were transfected with control, HO-1 siRNA or HO-1 plasmid for 36 hours and exposed to hemin for 12 hours. Western blotting analysis was then performed with antibodies against HO-1. (TIF) [file pone.0100677.s002.tif]

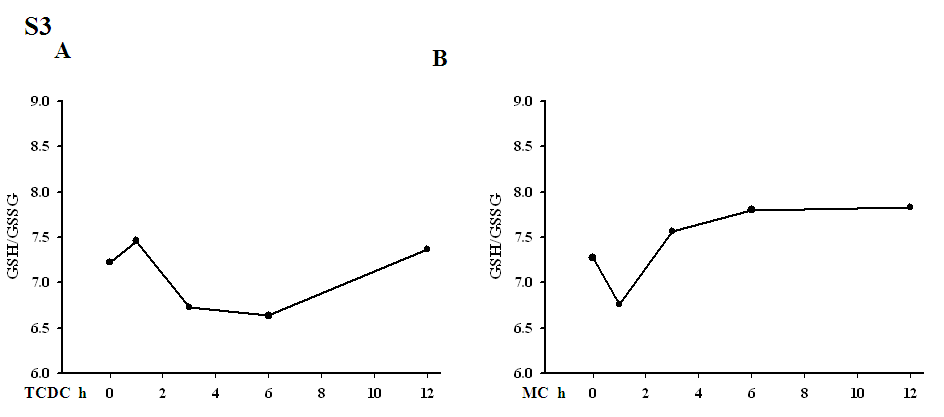

Supplement: Figure S3 — The GSH/GSSG ratio under CORMs treatment. A. and B. The GSH/GSSG ratio was determined at various time intervals. Results are presented as the mean from the data in Figure 4. (TIF) [file pone.0100677.s003.tif]

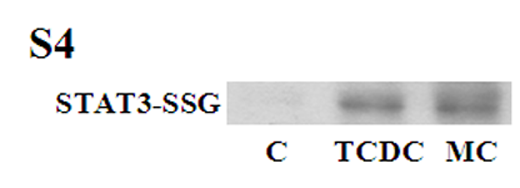

Supplement: Figure S4 — The detect of STAT3 glutathionylation. BAEC were loaded with biotin-labeled BioGEE (100 µmol/L, 1 h). Biotin-GSS tagged proteins were pulled-down with streptavidin-Sepharose beads and released with DTT (50 mmol/L), separated by SDS PAGE and immuno-blotted for STAT3. (TIF) [file pone.0100677.s004.tif]
